# Increased Risk of Genetic and Epigenetic Instability in Human Embryonic Stem Cells Associated with Specific Culture Conditions

**DOI:** 10.1371/journal.pone.0118307

**Published:** 2015-02-25

**Authors:** Ibon Garitaonandia, Hadar Amir, Francesca Sesillo Boscolo, Gerald K. Wambua, Heather L. Schultheisz, Karen Sabatini, Robert Morey, Shannon Waltz, Yu-Chieh Wang, Ha Tran, Trevor R. Leonardo, Kristopher Nazor, Ileana Slavin, Candace Lynch, Yingchun Li, Ronald Coleman, Irene Gallego Romero, Gulsah Altun, David Reynolds, Stephen Dalton, Mana Parast, Jeanne F. Loring, Louise C. Laurent

**Affiliations:** 1 Center for Regenerative Medicine, Department of Chemical Physiology, The Scripps Research Institute, 10550 North Torrey Pines Road, La Jolla, CA 92037, United States of America; 2 Department of Reproductive Medicine, UCSD Healthcare, 9500 Gilman Drive, Mail Code 0695, San Diego, CA 92093, United States of America; 3 Department of Pathology, UCSD Healthcare, 9500 Gilman Drive, Mail Code 0695, La Jolla, CA 92093-0612, United States of America; 4 Department of Human Genetics, University of Chicago, 920 E 58th St, CLSC 317, Chicago, IL, 60637, United States of America; 5 Department of Biochemistry and Molecular Biology, Center for Molecular Medicine, Paul D. Coverdell Center for Biomedical and Health Sciences, University of Georgia, Athens, GA, 30602, United States of America; Pohang University of Science and Technology (POSTECH), KOREA, REPUBLIC OF

## Abstract

The self-renewal and differentiation capacities of human pluripotent stem cells (hPSCs) make them a promising source of material for cell transplantation therapy, drug development, and studies of cellular differentiation and development. However, the large numbers of cells necessary for many of these applications require extensive expansion of hPSC cultures, a process that has been associated with genetic and epigenetic alterations. We have performed a combinatorial study on both hESCs and hiPSCs to compare the effects of enzymatic vs. mechanical passaging, and feeder-free vs. mouse embryonic fibroblast feeder substrate, on the genetic and epigenetic stability and the phenotypic characteristics of hPSCs. In extensive experiments involving over 100 continuous passages, we observed that both enzymatic passaging and feeder-free culture were associated with genetic instability, higher rates of cell proliferation, and persistence of OCT4/POU5F1-positive cells in teratomas, with enzymatic passaging having the stronger effect. In all combinations of culture conditions except for mechanical passaging on feeder layers, we noted recurrent deletions in the genomic region containing the tumor suppressor gene TP53, which was associated with decreased mRNA expression of TP53, as well as alterations in the expression of several downstream genes consistent with a decrease in the activity of the TP53 pathway. Among the hESC cultures, we also observed culture-associated variations in global gene expression and DNA methylation. The effects of enzymatic passaging and feeder-free conditions were also observed in hiPSC cultures. Our results highlight the need for careful assessment of the effects of culture conditions on cells intended for clinical therapies.

## Introduction

Human pluripotent stem cells (hPSCs, which include human embryonic stem cells (hESCs) and human induced pluripotent stem cells (hiPSCs)) hold great promise for clinical applications. Their tremendous self-renewal and differentiation capacities make them potential sources of large quantities of differentiated cells for drug screening, toxicology studies, biomolecule production, and cell therapy.

Perhaps the biggest area of concern in stem cell-based clinical transplantation is the possibility that the transplanted cells may be tumorigenic [1]. Since genetic and epigenetic instability have been strongly associated with cancers, it is reasonable to assume that such instability is undesirable in cell preparations destined for clinical use.

Large-scale genomic studies have revealed that both hESCs and hiPSCs accumulate subchromosomal genetic changes that are not detectable by karyotyping [2–6]. Much attention has been paid to the recurrent aberrations that have been found in multiple hPSC lines, including subchromosomal duplications on chromosomes 12 and 20, which are of particular interested because the chromosome 12 duplications frequently contain the pluripotency-associated NANOG gene and/or NANOGP1 (one of its pseudogenes), and the chromosome 20 duplication has been found to impart increased cell survival due to increased expression of Bcl-xL [7,8]. However, many sporadic genetic aberrations have been observed in only hPSC line, and the phenotypes of such aberrations have not been characterized.

Several small studies have indicated that time in culture or culture conditions may affect the genetic [9–11] or epigenetic [10] stability of hPSCs, suggesting that it will be important to future clinical applications to develop culture conditions that minimize acquisition of such abnormalities. Here, we report the first large-scale, systematic study of the potential effects of multiple culture conditions, including culture substrate, medium type, passaging method, and time in culture on the genetic, epigenetic and phenotypic stability of hPSCs.

## Materials and Methods

### Cell Culture

The HDF51iPS1, HDF51iPS7, and HDF51iPS11 lines were three independent hiPSC clones reprogrammed from human fetal dermal fibroblasts using the standard reprogramming factors (OCT4/POU5F1, SOX2, KLF4, and MYC) carried by retroviral vectors [3,12]. WA09 is an hESC line derived by the Thomson laboratory [13], and was obtained directly from WiCell Research Institute. WA09, HDF51iPS1, HDF51iPS7, and HDF51iPS11 cells were cultured at 37°C and 5% CO_2_ on extracellular matrix (Geltrex; Life Technologies) or irradiated mouse embryonic fibroblasts (MEFs). Cells on feeder layers were cultured in standard hPSC medium, consisting of Dulbecco’s modified eagle medium DMEM/F12 (Life Technologies) with 20% Knockout Serum Replacement (Life Technologies), 1 mM GlutaMAX (Life Technologies), 0.1 mM non-essential amino acids (NEAA, Life Technologies), and 12 ng/ml basic FGF (Life Technologies) and passaged mechanically (MefMech) by cutting colonies into small pieces with a 18G needle or enzymatically dissociated (MefEnz) with Accutase (Life Technologies). Cells cultured on Geltrex in StemPro hESC SFM (Life Technologies) with 0.1 mM β-mercaptoethanol (Life Technologies) and 12 ng/ml basic FGF (Life Technologies), were passaged mechanically (EcmMech) or enzymatically (EcmEnz) with Accutase. Cells on feeder layers were passaged once a week and feeder-free cultures were passaged every three to four days. At each passage, 150,000 cells were seeded per well of a 6-well plate (equivalent to a cell plating density of ~15,600 cells/cm^2^). To accurately count the number of cells in mechanically passaged cultures, the clumps of cells were collected and gently triturated, and an aliquot was removed and completely dissociated to single cell suspension using Accutase. The medium was changed daily.

In transitioning the original cell cultures from the MefMech condition into the other conditions, we started counting the passages in the new condition as soon as the process of transition started. For transitioning from mechanical to enzymatic passaging, we simply started using Accutase (Life Technologies). For transitioning from the Mef condition (which included culture on mouse embryonic fibroblast feeder layers with the standard hPSC medium) to the Ecm condition (which included culture on Geltrex (Life Technologies) with StemPro hESC SFM (Life Technologies)), we initially passaged onto Geltrex (Life Technologies) using a 1:1 mixture of standard hPSC medium:StemPro hESC SFM for 24 hours, followed by a 1:3 mixture of standard hPSC medium:StemPro hESC SFM for 24 hours, and then 100% StemPro hESC SFM thereafter.

### Teratoma formation, histopathologic evaluation and immunohistochemistry

All procedures were done following the National Institute of Health (NIH) Guide for the Care and Use of Laboratory Animals and were approved by the Scripps Research Institute and UCSD Institutional Animal Care and Use Committees (IACUCs). For the teratoma assays, we injected two mice per replicate (12 mice per culture condition) at two sites per mouse (1 testis capsule and 1 leg injection). WA09 hESCs were harvested by Accutase (Life Technologies), washed with PBS (Life Technologies), re-suspended in 30 μl of a 1:1 mixture of DMEM/F12 (Life Technologies) and Matrigel (BD Biosciences), and placed on ice. One million cells were injected per site into the right testis capsule and left lower leg muscle of CB-17-Prkdcscid mice (Charles River) with a 1cc syringe and a 27G, ½ inch needle. The injection site was monitored for tumor growth weekly and six to eight weeks after injection the mice were euthanized and the tumors removed. The tumors were measured, weighed, and fixed with PBS containing 4% paraformaldehyde (Sigma-Aldrich). The specimens were sectioned and stained with hematoxylin and eosin and evaluated microscopically by a board-certified anatomic pathologist (MP) to detect derivatives of the three germ layers. Selected sections were stained with a rabbit anti-OCT4 primary antibody (Abcam, Cat # ab19857) at 1:400, using a Ventana Discovery Ultra automated immunostainer (Ventana Medical Systems) with standard antigen retrieval and reagents per the manufacturer’s protocol.

### Karyotyping

Karyotyping was performed only on selected MefEnz cultures. Cell Line Genetics performed G-banded karyotyping on the hESC cultures. 30 spreads were counted per culture.

### Mycoplasma Testing

Supernatant of the cultures was collected and mycoplasma was tested monthly using the Lonza Luminescence test (Lonza) and VenorGeM PRC Test (Sigma-Aldrich) according to the manufacturers’ protocols.

### *In Vitro* Differentiation

For embryoid body (EB) formation, WA09 hESCs were harvested manually and transferred to low attachment plates in Dulbecco’s modified eagle medium DMEM/F12 with 20% Knockout Serum Replacement, 1 mM Glutamax, 0.1 mM non-essential amino acids (NEAA), 0.1 mM β-mercaptoethanol. The cells were cultured in suspension for 7 days to allow for EB formation and the media was changed every other day. On the eighth day, the EBs were transferred to gelatin (Life Technologies) coated coverslips with DMEM (Life Technologies), 2 mM Glutamax, 20% fetal bovine serum (Life Technologies), and 0.1 mM NEAA, and cultured seven more days changing the medium every other day. EBs were then washed with PBS and fixed for 15 minutes with PBS containing 4% paraformaldehyde.

### Immunocytochemistry

Cells were fixed with 4% paraformaldehyde in PBS for 15 minutes at room temperature. After washing with PBS, the cells were treated with blocking solution (2% BSA (Sigma-Aldrich), 0.1% Triton X-100 (Sigma-Aldrich), and 2% low fat milk in PBS) for one hour at room temperature. Primary antibodies include OCT4 (1:100, Santa Cruz); SSEA-4 (1:200, R&D system); SOX2 (1:100, Millipore); TUJ1 (1:15,000, Millipore); Brachyury (1:300, Santa Cruz); Smooth Muscle Actin (SMA) (1:2000, Millipore); alpha-fetoprotein (AFP) (1:400, Millipore). Fluorescent dye-labeled goat anti mouse, goat anti rat or donkey anti mouse was used as the secondary antibody (Life Technology, Molecular Probes).

### EDU Incorporation Assay

Cells were incubated with 20 μM EdU (Life Technologies) for 1 hour at 37°C, harvested, washed with 1% BSA in PBS, and fixed in suspension for 15 minutes at room temperature with 4% paraformaldehyde. Cells were washed again, permeabilized with Triton X-100 for 30 minutes at room temperature, washed again, and incubated with the Click-iT EDU CellCycle 633-red (Life Technologies) for 30 minutes at room temperature and protected from light. The samples were then analyzed by flow cytometry.

### MTT Assay

Cells cultured on a 96-well plate were incubated in medium containing 0.4 mg/mL 3-[4,5-dimethyl-thiazol-2-yl]-2,5-diphenyl-2H-tetrazolium bromide (MTT; TCI America) at 37°C for 1 h. Reduced MTT was solubilized in DMSO (Sigma-Aldrich) for determination of absorbance at 570 nm.

### Apoptosis Assay

Apoptosis rate was measured using the In Situ Cell Death Detection Kit (Roche). Cells were harvested and a cell suspension of 10^6^ cells/ 100 μl was fixed with 4% paraformaldehyde for 60 min at room temperature with shaking, washed with PBS, and permeabilized with 0.1% Triton X-100 in 0.1% sodium citrate (Sigma-Aldrich) for 2 minutes on ice. The labeling of DNA strand breaks was performed for 60 minutes at 37°C by Terminal deoxynucleotidyltransferase (TdT), which catalyzes polymerization of fluorescein labeled dUTP to free 3’-OH DNA ends in a template independent manner, also known as TUNEL reaction. The cells were then washed with PBS and analyzed by flow cytometry at an excitation wavelength of 488 nm and detection at 515–565 nm.

### Telomere Length Assay

Telomere length was measured using TeloTAGGG Telomere length assay (Roche). Cells were harvested and genomic DNA was isolated with DNeasy Blood & Tissue kit (Qiagen). The concentration of genomic DNA was measured with the Qubit Quantitation Platform (Invitrogen), the DNA concentration for each sample was normalized, and 1.5 μg of genomic DNA from the sample or control DNA was digested with 40 units of Hinf I (New England Biolabs) and Rsa I (New England Biolabs) for 2 hours at 37°C. The digestion was stopped with gel electrophoresis loading buffer and the digested DNA was separated by 0.8% agarose gel electrophoresis. For Southern blots, the digested DNA was transferred to a positively charged nylon membrane (Roche) using capillary transfer. The DNA was fixed on the wet blotting membrane by UV-crosslinking at 120 mJ. The blotted DNA fragments were then hybridized to a digoxigenin (DIG)-labeled probe specific for telomeric repeats for 3 hours at 42°C, washed several times, and incubated for 30 minutes at room temperature with a DIG-specific antibody covalently coupled to alkaline phosphatase. The membrane was incubated with the chemiluminescent substrate CDP-Star and imaged (Gel Doc XR System, Bio-Rad). The mean terminal restriction fragment (TRF) length, which comprises the variable terminal array and the subtelomeric region, was calculated by measuring the chemiluminescent signal and the length of each TRF.

### Telomerase Activity Assay

Telomerase activity was measured using TRAPeze RT Telomerase Detection kit (Millipore). Cells were harvested, washed with PBS, resuspended in 200 μl of CHAPS lysis buffer (provided in TRAPeze kit)/10^6^ cells, and incubated on ice for 30 minutes. The samples were spun down at 12,000 x g for 20 minutes at 4°C, 160 μl of supernatant was collected, protein concentration determined with the BCA Protein Assay (Pierce), and the concentration of total protein was normalized for each sample. The reaction mixture contained 5 μl 5X TRAPeze RT Reaction Mix, 1 μl Taq polymerase (Titanium Taq, Clontech #639220), 17 μl nuclease free water, and 2 μl of TSR8 dilution (for standard curve), positive extract control, telomerase negative control, no template control, experimental samples, or heat treated experimental samples. The parameters for the qRT-PCR were 30 minutes at 30°C (1 cycle), 2 minutes at 95°C (1 cycle), and then15 s at 94°C, 60 s at 59°C, 10 s at 45°C for 45 cycles using the primers are supplied by the manufacturer.

### SNP Genotyping

SNP Genotyping was performed on the OmniQuad version 1 (Illumina, Inc.), which interrogates 1,140,419 SNPs across the human genome. One μg input genomic DNA purified with the DNeasy Blood & Tissue Kit (Qiagen) (the yield from approximately 200,000 cells) was amplified and labeled according to the manufacturer’s instructions. DNA was quantified using the PicoGreen reagent (Life Technologies). Labeled product was then hybridized to the array and scanned on a BeadArray Reader (Illumina, Inc.). Genotyping calls were made using BeadStudio (Illumina, Inc.), using the standard cluster files provided by the manufacturer. The GenCall threshold was set to 0.15, and the call rates were between 0.9911858 (EcmMech P147 D) and 0.9981838 (Control).

### Copy Number Variation Assessment

For the SNP Genotyping data, data preprocessing was performed in BeadScan (Illumina, Inc.). Data cleaning, SNP calling, and replicate error identification was performed in GenomeStudio (Illumina, Inc.). CNVPartition 3.2.0 [14] was used as the CNV-calling algorithm for calling duplications, with a CNV score threshold set at 50, and a minimum number of 10 SNPs per CNV region. CNV locations are displayed according to the hg19/NCBI36 version of the human genome. We have previously shown that approximately 80% of the duplications called by this method are validated by qPCR [3]. Since we previously found that only 40% of the regions of deletion identified by CNVPartition were validated by qPCR [3], we required that regions of deletion be identified both as regions of deletion by CNVPartition and as new regions of LOH by replicate error analysis. All CNV calls were manually curated, and regions that were identified manually are indicated with a CNV Confidence Value designated as “NA”. Manual curation was also used to combine adjacent areas called by CNVPartition into larger regions of CNV; in these cases, the CNV confidence interval is indicated as the range of CNV confidence intervals calculated by CNVPartition for the encompassed subregions. During manual curation, we identified some regions of CNV that were present in only a subpopulation of cells, which we termed “partial” duplications and deletions. We also identified some large regions of partial CNV for which we could not determine with certainty whether they were partial duplications or partial deletions, and these we termed “Complex CNV.”

### CNV-RT-PCR

Validation of the CNV values, obtained from CNVPartition 3.2.0., was done using TaqMan Copy Number Assays (Life Technologies). The DNA used for validation was the same on which SNP genotyping analysis was performed. The DNA was diluted in order to obtain a final concentration of 5 ng/μl. 20 ng of DNA were loaded in the plate. Following the manufacturer’s protocol, we prepared the reaction mixture (10 μl of TaqMan Genotyping Master Mix (Life Technologies), 1 μl of probe of choice (Life Technologies), 1 μl of TaqMan Copy Number Reference Assay (Life Technologies), and 4 μl of nuclease free water) and added to each sample. qPCR was performed using these cycling conditions: 10 minutes at 95°C (1 cycle), and then 15 s at 95°C, 60 s at 60°C, for 40 cycles. Six probes (three for the short arm and three for the long arm of chromosome 12, Life Technologies)) were used to validate the CNV values of the samples: Hs03295596_cn (12p11.22b chr12:29111191), Hs04404518_cn (12p11.21b ch12:31250144), Hs03812366_cn (12p11.21b chr12:31944796), Hs03841181_cn (12q23.1b chr12:979244490), Hs06947059_cn (12q24.11d chr12:111495082), and Hs06363301_cn (12q24.23a chr12:118819770). The results were analyzed using the ΔΔCt method, using RNaseP as the control.

### Gene Expression Microarray

Gene expression profiling was performed using HumanHT-12 v3 Expression BeadChips according to the manufacturer’s instructions (Illumina, Inc.), with the exception of the definitive mesoderm sample, for which gene expression profiling was performed using HumanHT-12 v4 BeadChips (Illumina, Inc.). Samples were prepared using the TotalPrep kit (Ambion, Inc.) according to the manufacturer’s instructions. Probes were filtered with a detection-*P* value cut-off of 0.05, and normalized using the LUMI package in R with the RSN (Robust spline normalization) method.

Multivariate analysis was performed on all the undifferentiated samples using R. Different variables were compared: time in culture (early vs. late), passage method (Enz vs. Mech), and substrate (Mef vs. Ecm). The output was filtered with a p-value cutoff of <0.01 and a fold-change cutoff of >1.8.

Hierarchical clustering analysis was performed to determine the relative expression levels of the pluripotency-associated genes using Cluster 3.0. The dendrograms and heatmaps were generated using Java Treeview [15].

Pathways analysis was performed with DAVID Bioinformatics Resources 6.7 [16].

### DNA Methylation Microarray

DNA was extracted from 10^6^ cells (Qiagen DNeasy kit) according to the manufacturer’s protocol and quantified using the Picogreen reagent (Life Technologies). Bisulfite conversion was performed using the Bisulfite Conversion Kit from Zymo Research. Bisulfite converted DNA was labeled and hybridized to Infinium HumanMethylation450 BeadChips (Illumina, Inc.), scanned with an Illumina iScan BeadArray Scanner and quality controlled in GenomeStudio (Illumina, Inc.). Pathway analysis was performed using GREAT [17], and visualized using REVIGO [18].

### Accession Numbers

The hESC DNA methylation, gene expression, and SNP genotyping array data are available at the NCBI GEO database under the accession designation GSE34982. The hiPSC SNP genotyping data are available under accession number GSE56834. The hESC and hiPSC DNA methylation data are also available under SuperSeries GSE56851.

## Results

We designed a highly replicated, combinatorial experiment to determine the effects of several culture parameters on the long-term stability of hESC and hiPSC cultures. For the hESC experiments, we received cryopreserved WA09 hESCs in WiCell medium at passage 28. The WA09 hESCs were derived (the first 6 passages) on mouse embryonic fibroblast (MEF) feeder layers in basal medium with fetal bovine serum and human LIF; this was, followed by 22 passages in standard hESC medium on MEFs. All passaging was done with collagenase [13].

**Fig 1:**
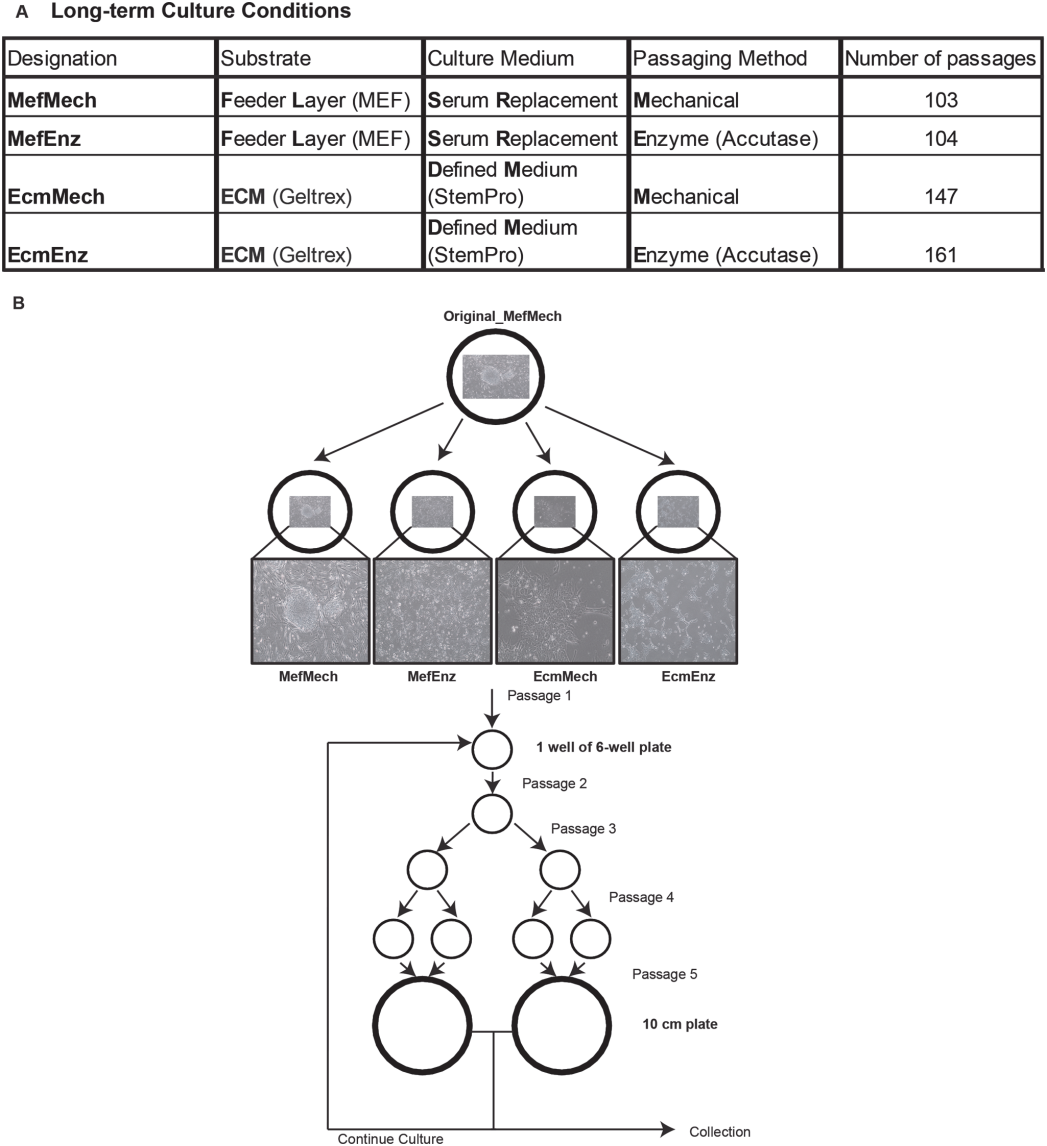
Experimental design. **A**. Description of the four culture conditions used for the expansion of hESC WA09. **B**. Culture expansion and sample collection strategy.

After thawing the cells onto MEFs in standard hESC medium and passaging them mechanically for 9 passages, the resulting passage 37 cells were used as the starting population of cells for this study. We cultured the hESCs continuously for more than 100 additional passages (nearly two years) in four different conditions, with six replicates per condition (Fig. 1 and S1 Table), which allowed us to distinguish the effects of two types of media (hESC medium with Serum Replacement (see Materials and Methods) and defined medium (StemPro; Life Technologies, Inc.)), two substrates (feeder layer and extracellular matrix (Geltrex; Life Technologies, Inc.)), and two passaging methods (mechanical passage and enzymatic passage (Accutase; Life Technologies, Inc.)). The passage numbers used throughout this manuscript are the number of passages after switching to the new culture conditions.

Each hESC culture was cryopreserved, tested for mycoplasma, and collected for molecular analysis every 5 passages (Fig. 1B, S1 Table). Representative images showing typical cell morphology in each culture condition were taken 5 passages after transitioning the cells into the four different conditions (Fig. 1B). This was done shortly after placing the cells into the new conditions to minimize the chance that differences in morphology would be due to genetic changes. The hESCs cultured on MEF feeder layers with mechanical passaging (MefMech) grew in large colonies with well-defined borders, while the hESCs on MEF feeder layers with enzymatic passaging (MefEnz) grew in significantly smaller colonies, due to the fact that the enzymatic passaging separated the cells into a single cell suspension. The hESCs cultured on a cell-free extracellular matrix grew in irregular clusters without defined borders (in contrast to the cells cultured on MEFs), with the mechanically passaged cultures (EcmMech) having larger clusters compared to the enzymatically passaged cultures (EcmEnz), consistent with the enzymatic dispersion of the cells into a single-cell suspension. Samples at selected passage numbers were analyzed for both global gene expression and SNP genotyping to assess genomic stability, and DNA methylation profiling to monitor epigenetic stability. Cells in each condition were also extensively phenotyped, including assays for cell proliferation, telomerase activity, apoptosis, pluripotency markers and differentiation capacity. These data have allowed us to determine how differences in passage method, substrate, or medium type affect the hESCs.

For the hiPSC studies, the initial clones for the HDF51iPS1, HDF51iPS7, and HDF51iPS11 lines were first manually picked onto MEFs in standard hPSC medium and passaged mechanically for 5–8 passages. We then sub-cultured each hiPSC line, maintaining one sub-culture in the MefMech condition, and adapting one sub-culture to the EcmEnz condition. The cultures were then passaged for an additional 25–35 passages in the two different conditions. The initial cultures at passage 5–8, and the final cultures collected 25–35 passages later in the two different conditions were analyzed using SNP genotyping with CNV calling to assess genomic stability.

### Genetic Stability

For each of the four cell lines included in this study (one hESC line and three hiPSC lines), we started with a single culture, and divided it into sub-cultures that were then carried in parallel in different culture conditions (Fig. 1B). In this way, the cells from the original cultures could serve as a baseline to identify genetic changes that occurred over the course of subsequent long-term passage in the different culture conditions. SNP Genotyping and CNV calling were performed on the original cultures to identify CNVs present at the start of the experiment (S2A-B Table, “Original_MefMech” for the WA09 hESC line; S2C-E Table, “HDF51iPS1 Original_MefMech_P6”, “HDF51iPS7 Original_MefMech_P5”, “HDF51iPS11 Original_MefMech_P8”). The CNVs identified in the original cultures were subtracted from the results from all later cultures, which allowed us to specifically identify *de novo* CNVs that arose during the course of the experiment.

For the WA09 hESC line, the first round of SNP genotyping was performed on the original culture, and cells cultured in the four conditions for ~40 passages and 103–161 passages beyond the initial 37 passages. For a subset of conditions, we performed additional SNP genotyping to better delineate the passage number at which *de novo* CNVs arose (S1 Table).

For the three hiPSC lines, SNP genotyping was performed on the initial passage 5–8 cultures, and the MefMech and EcmEnz cultures collected 25–33 passages later.

### Differences in Numbers of Genetic Aberrations in Different Conditions

**Fig 2:**
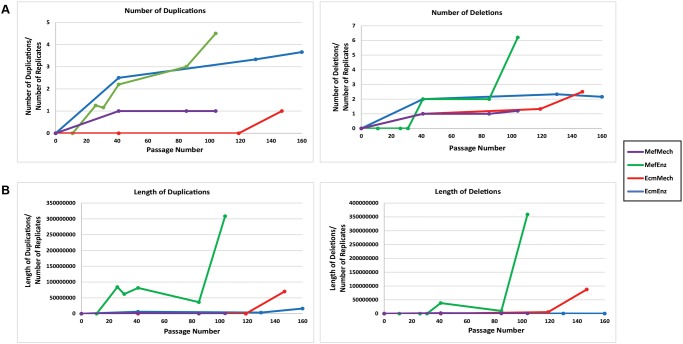
Graphs illustrating the number (A) and total length (B) of genetic aberrations. Aberrations were identified in the samples using CNV analysis of high resolution SNP genotyping data from the WA09 hESC line. Duplications and deletions; regions of loss of homozygosity (LOH) were categorized as deletions and complex CNVs were categorized as duplications. The x-axis indicates the number of passages for the current study. All cultures started from a source culture that had been passaged for 37 passages. Genetic aberrations present in this source culture were not counted, and therefore at the start of the study, the number of duplications and deletions were set at 0. Of note, each culture condition was assigned a unique color that is consistent throughout the manuscript.

**Fig 3:**
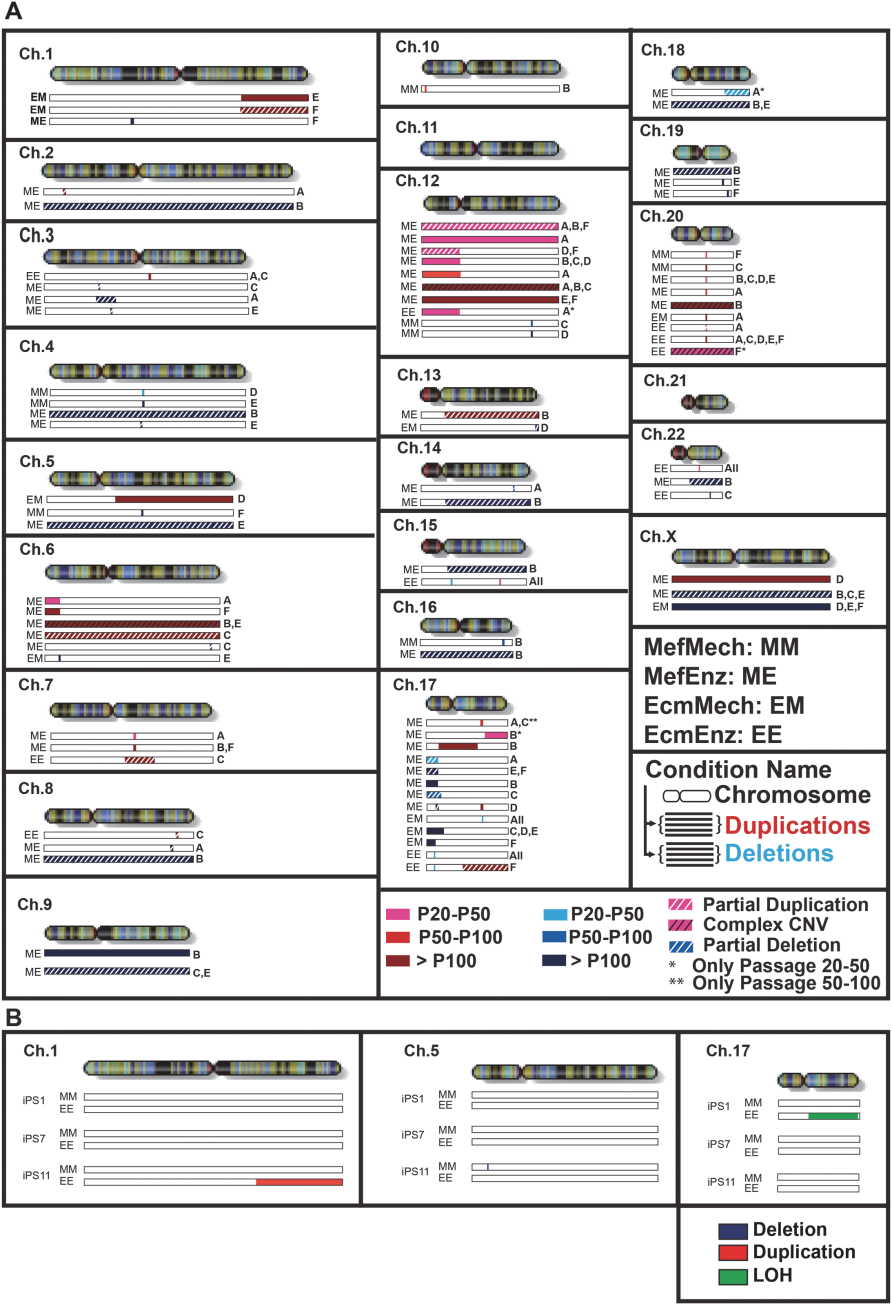
Overview of genetic aberrations. Results are shown for the WA09 hESC line (**A**) and the HDF51IPS1, HDF51IPS7, and HDF51IPS11 hiPSC lines (**B**). Fig. 3A shows regions of duplication and deletion in the 4culture conditions for the WA09 hESC line. Duplications (3 or 4 copies) (identified by CNVPartition 3.2.0) are shown below the chromosome in a red scale. Deletions (0 or 1 copy) (identified by CNVPartition 3.2.0 and replicate error analysis) are shown below the regions of duplication in a blue scale. Red and blue scales are used to represent the passage when the event (duplication or deletion) first occurred, and they range from light colors (early passages) to darker colors (late passages). White and black hatches represent partial mutations (duplications or deletions that are present in only a subpopulation of cells) and complex CNVs (CNVs that appear to be composed of both duplicated and deleted regions, for which the precise copy numbers cannot confidently be called based on the SNP genotyping data), respectively. Asterisks represent mutations that were present only in earlier passages and were not detectable in later passages. For each CNV in each condition, the replicate(s) in which the event was detected is indicated by a letter to the right of each bar. Fig. 3B shows regions of duplication, deletion and LOH in the 2 culture conditions for the hiPSC lines. Duplications are shown in blue, deletions are shown in red and regions of LOH are shown in green. The chromosome ideograms are from http://genomics.energy.gov.

In the hESC cultures, we compared the total number (Fig. 2A) and length (Fig. 2B) of duplications and deletions in each of the four culture conditions over time (see also Fig. 3A), as determined by SNP genotyping. Larger numbers of duplications were seen in the enzymatic passage conditions (MefEnz and EcmEnz), suggesting that dissociation to single cells during passaging might favor the accumulation of duplications. Between these two conditions, the MefEnz appeared to be associated with larger duplications (Fig. 2B). In contrast, the mechanical passaging conditions, MefMech and EcmMech, showed low numbers of duplications, most of which arose at very late passage. The same trends held for deletions, with the MefEnz and EcmEnz conditions showing higher frequencies of deletions, and the MefEnz condition showing the largest number of deletions at higher passages. These data indicate that Accutase-based enzymatic passaging is associated with higher rates of genetic instability.

For the hiPSC studies, we focused on two conditions, MefMech and EcmEnz, and performed our studies on one replicate each for three different hiPSC lines (Fig. 3B and S2C-E Table). Two of the three hiPSC lines, HDF51iPS1 and HDF51iPS11, acquired a large aberration in the EcmEnz condition. In HDF51iPS1, we observed a large region of LOH (loss of heterozygosity) on the long arm of chromosome 17, and in the HDF51iPS11 line, we observed a large duplication on the short arm of chromosome 1. A small deletion developed in the HDF51iPS11 line in the MefMech condition (Fig. 3B). This deletion encompassed 2 piRNAs for which no specific functions have been identified (hsa_piR_005610 and hsa_piR_021822), no identified protein-coding genes, no sequences marked by H3K27Ac (associated with active enhancer sequences) in the WA01 hESC line in the ENCODE dataset (genome.ucsc.edu/ENCODE/downloads.html), and no experimentally validated enhancer sequences listed in the VISTA database (enhancer.lbl.gov). Although the hiPSC experiments were performed over a relatively small number of passages (25–33 passages) and the total number of aberrations that arose during the course of experiment was small, the hiPSC results are consistent with the hESC results, which indicate that cells cultured in the EcmEnz condition accumulate larger genetic aberrations at a higher frequency than those cultured in MefMech conditions.

### Recurrent Duplications on Chromosomes 12 and 20 in the hESC cultures

**Fig 4:**
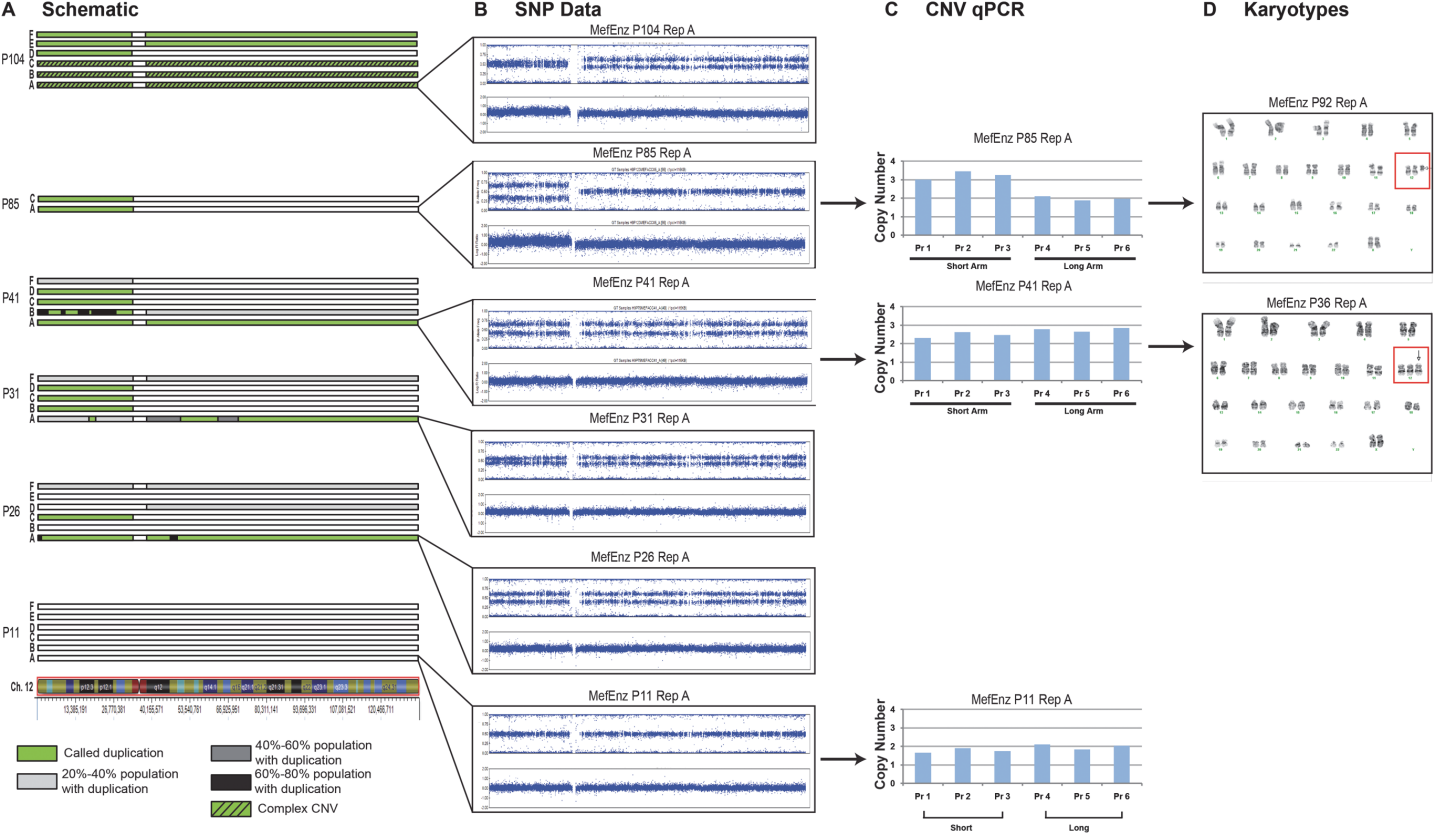
Chromosome 12 detail in MefEnz. Detailed view of the duplications in chromosome 12 for MefEnz. **A**. The green areas indicate the regions of duplication that were identified by CNVPartition 3.2.0. The areas that were detected manually rather than by the software are indicated in a scale from grey to black. Estimates of the percentage of the population carrying the duplication were performed using the BAF distance for heterozygous SNPs as described [3]. **B**. LogR Ratio (LRR) and BAF plots of replicate A of MefEnz showed changes in genetic aberrations over time. **C**. The CNV data were further validated via TaqMan Copy Number Assays. Probe 1 corresponds to Hs03295596_cn, Probe 2 to Hs04404518_cn, Probe 3 to Hs03812366_cn, Probe 4 to Hs03841181_cn, Probe 5 to Hs06947059_cn, and Probe 6 to Hs06363301_cn. **D**. Karyotyping results confirmed the CNV data.

An overview of the regions of CNV that arose in the WA09 hESC line when cultured in different conditions is shown in Fig. 3A (S2A-B Table). Since we acquired data at multiple time points over the course of the experiment, we were able to follow the accumulation of genetic aberrations. Most dramatically, we observed a duplication of a small segment of chromosome 20 in multiple cultures, which we have previously described as a region of recurrent duplication among a large collection of hPSCs [3]. This duplication was observed after passage 40 in at least one of the replicates in all of the conditions. Since the same duplication (with the same start and end locations) appeared in multiple replicates in more than one condition, it is likely that there was a small subpopulation of cells carrying this duplication within the original cell population that was undetectable by our analysis methods. We also observed duplications on chromosome 12 in the MefEnz condition (Fig. 3A; Fig. 4; S2A Table); recurrent duplications of all or part this chromosome in hPSCs have also been described in other studies [3,19].

### Recurrent Deletions on Chromosomes 17 Encompassing the TP53 gene in the hESC cultures

We also observed the accumulation of deletions, including deletions that arose in more than one replicate of more than one condition (Fig. 3A; S2 Table). For the MefMech, EcmMech, and EcmEnz conditions, the deletions appeared to be quite small, were detected after 40 passages, and remained unchanged for the remainder of the experiment. For the MefEnz condition, the number and length of deletions appeared to increase again in later passages.

**Fig 5:**
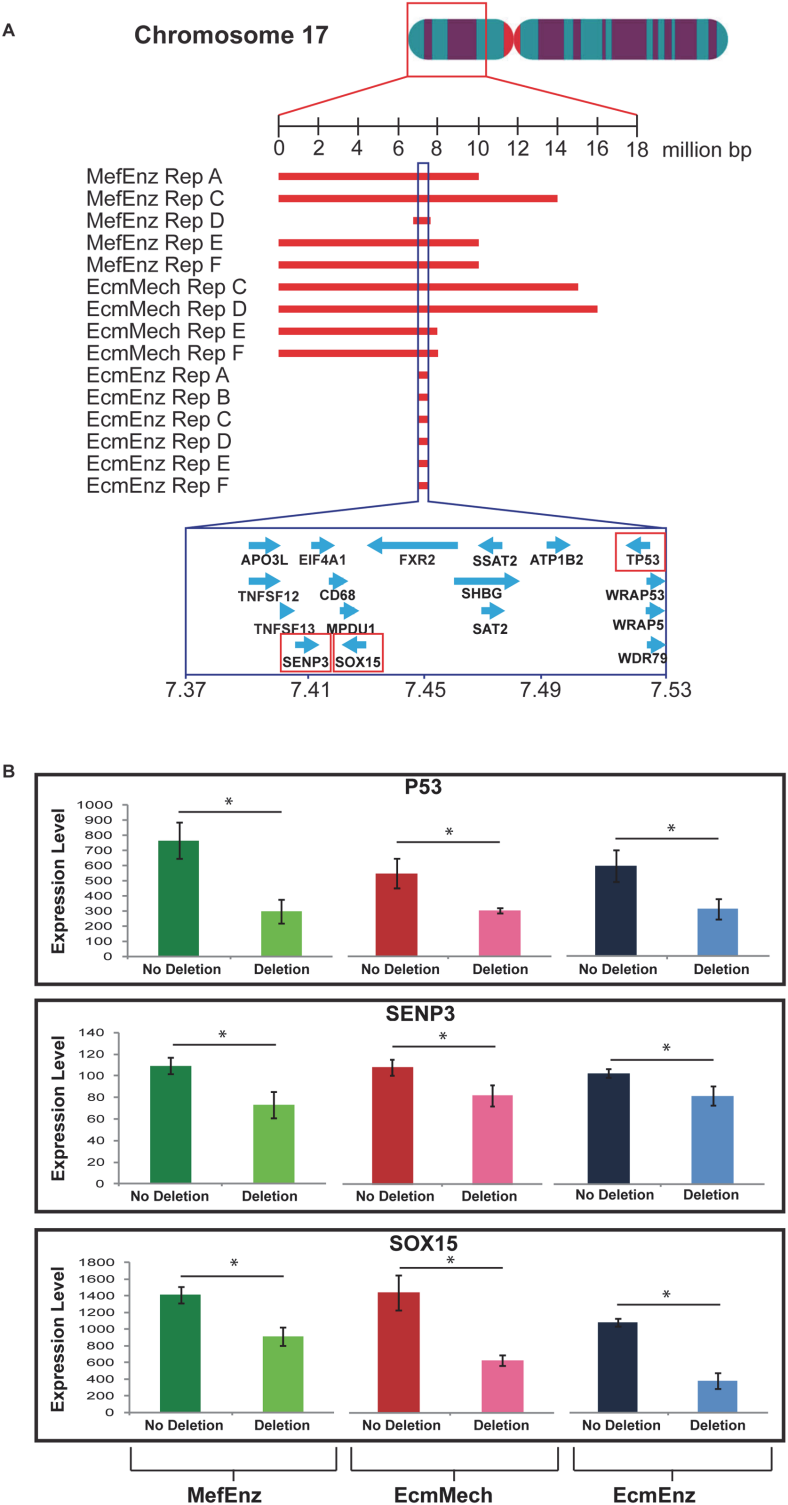
Detail of recurrent deletions in chromosome 17. **A**. The short arm of the chromosome is enlarged and the regions that showed deletions in the MefEnz, EcmMech, and EcmEnz conditions are indicated by red bars. Blue lines enclose the common area among all the conditions and the genes in the common area are indicated in the lower part of the figure. Genes for which the level of expression correlated with copy number are highlighted by red squares. **B**. Graphs representing the expression levels of TP53, SENP3, and SOX15, as measured by gene expression microarray. The ideogram of the chromosome was from the U.S. Department of Energy Genome Programs.

Most strikingly, there were recurrent deletions on the short arm of chromosome 17 in four replicates of MefEnz (at passage 41 for one replicate and at passage 104 for three replicates), four replicates of EcmMech (at passage 147), and all six replicates of EcmEnz (at passage 41). These deletions all overlapped within a 158 kb region (Fig. 5A), and were associated with decreased expression of three of the 17 genes located in this region, as measured using HumanHT-12 BeadChip microarrays (Illumina). These three genes were *SENP3* (SUMO1/sentrin/SMT3 specific peptidase 3), *SOX15* (SRY-BOX 15), and *TP53* (P53), a well-known tumor suppressor gene (Fig. 5B) [20]. Gene expression data for all of the genes in the deleted region are shown in S1 Fig. (please note that some probes are located in regions where two or three genes overlap, and that there are two probes each for CD68 and MPDU1).

It is notable that many of the observed deletions encompassing the *TP53* gene had different start/end positions, even among replicates of the same culture conditions (Fig. 3A; Fig. 5). This suggests that several deletion events in this region arose independently and underwent positive selection during the study.

### Dynamic Genetic Aberrations over Time in Culture in the hESC Cultures

In a prior study, we observed dynamic shifts in subpopulations of cells within a culture, in which one subpopulation was genetically normal and other subpopulations carried a given CNV [3]. In this study, we again observed apparent gains and losses of specific genetic aberrations over time in several of the hESC cultures. For example, in replicate A of the MefEnz condition, a complete duplication of chromosome 12 appeared at passage 26, and was present at passages 31 and 41, as detected by CNV analysis of SNP genotyping data (Fig. 4B). As verification of the SNP genotyping results, we performed a qRT-PCR-based CNV assay on stored DNA from the passage 41 culture (Fig. 4C), and thawed viable cryopreserved cells from passage 31, and after recovery from the thawing process performed karyotyping [47, XX, +12] at passage 36 (Fig. 4D). Between passages 41 and 85, the long arm was deleted and the short arm was duplicated from one of the three copies of chromosome 12, resulting in the cells carrying two copies of the long arm and four copies of the short arm of chromosome 12 (confirmed by qRT-PCR on stored DNA from the passage 85 culture and karyotyping of cells cryopreserved at passage 85 and recovered to passage 92 [47, XX, +i(12)(p10)]). Between passages 85 and 104, the pattern again changed, this time to a complex CNV that is difficult to characterize based on the BAF and LRR plots alone. We believe that this complex pattern arises from a mixed population of cells, in which there are multiple subpopulations with different numerical abnormalities for the p and q arms of chromosome 12.

In another example, we observed that a duplication of the short arm of chr12 in EcmEnz culture A was seen in samples collected between passage 20 and passage 50, and then seemed to disappear from the culture by passage 120 (Fig. 3A). As in our previous study [3], we attribute this to mosaicism within cell cultures, which can result in changes of the proportion of cells that carry a specific CNV over time. Since we analyzed bulk populations of cells, our results reflect the predominant state of the culture, rather than the individual cells in the culture.

### Assays to Identify Phenotypic Differences in hESCs Associated with Culture Conditions

To determine whether genomic differences would affect hESCs at the phenotypic level, we carried out a set of assays aimed at quantifying cell proliferation, cell cycle distribution, telomerase activity, telomere length, apoptosis, pluripotency, and differentiation capacity, after at least 50 passages in the assigned culture conditions (S1 Table). In contrast to the genotyping results, which revealed clear patterns of recurrent genetic aberrations that corresponded to specific culture conditions, the results of the phenotypic assays were subtle.

**Fig 6:**
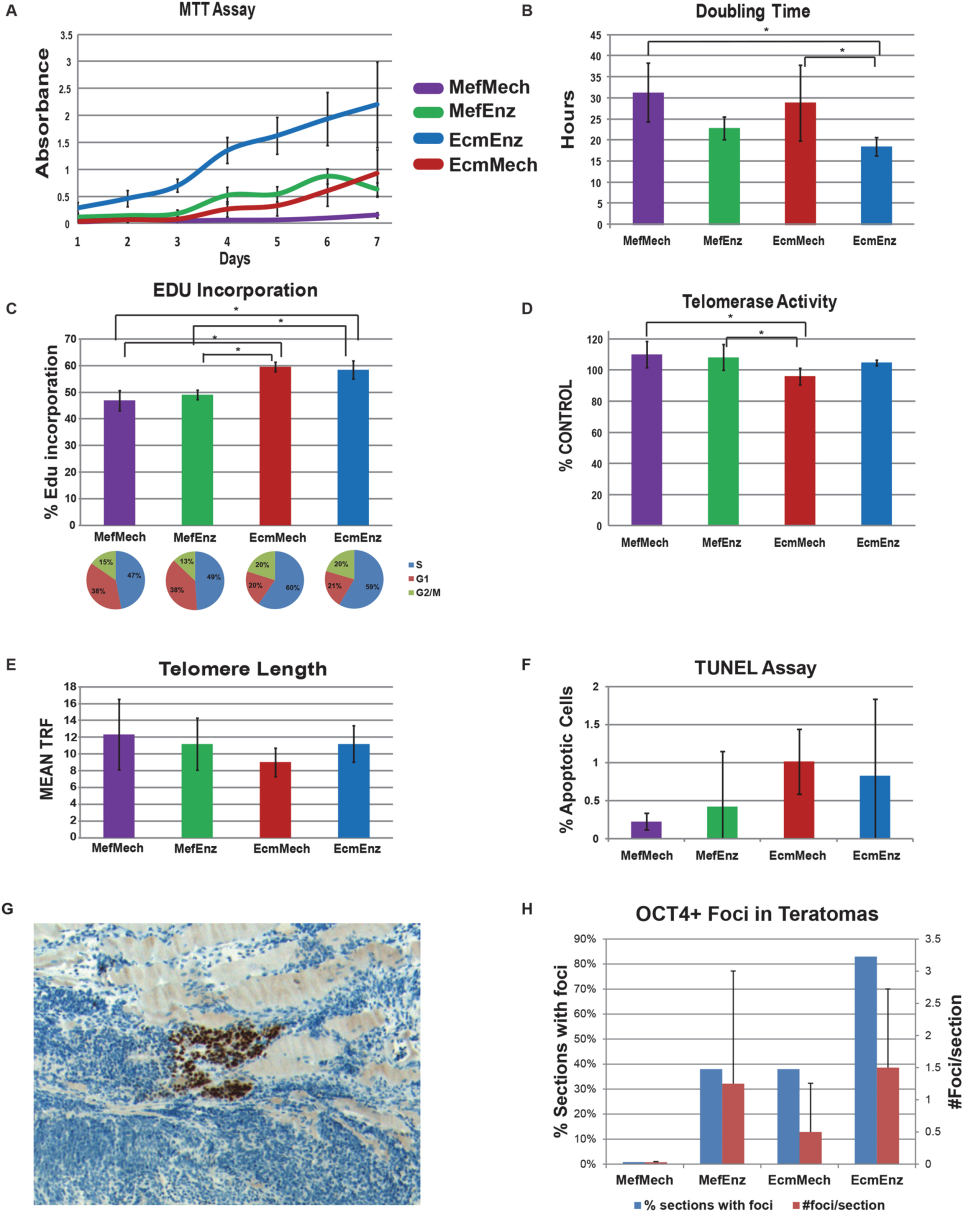
Phenotypic assays. Horizontal brackets indicate significant differences between the specified conditions, as calculated using ANOVA (p-value<0.05). **A**. The MTT assay measures cell proliferation, with absorbance being correlated with cell growth. **B**. Doubling time was calculated from cell counts taken over 3 days (average of 3 replicates per condition). **C**. The EdU incorporation was determined via FACS, and reveals the cell cycle distribution. The graph shows the percent of cells in S phase. **D**. Relative telomerase activity is shown. **E**. Average number of telomere repeat fragments (TRF, average of 3 replicates) for each condition. **F**. Percent of apoptotic cells, determined using TUNEL-FACS. **G**. Example of an OCT4-positive focus in a teratoma section. **H**. Bar graph showing, for each culture condition, the percent of teratoma sections containing at least one OCT4-positive focus, and the average number of foci per section. The error bar indicates the standard deviation of counts from 6 (EcmEnz), 7 (MefMech), or 8 (MefEnz and EcmMech) sections. Raw counts can be found in S4 Table).

To assess cell proliferation, we used the MTT assay, in addition to calculating doubling times. Both assays indicated that enzymatic passaging was associated with higher proliferation rates (Fig. 6A-B; S3 Table). Cell cycle distribution was determined by EdU incorporation and quantitative DNA staining with Pacific Blue, followed by FACS analysis (Fig. 6C; S3 Table). hESCs grown in the feeder-free conditions showed a higher level of EdU incorporation and a larger proportion of cells in S phase (p<0.05).

Telomerase activity was quantified using the TRAP assay and telomere length was measured by Southern blot using a digoxigenin-labeled probe (TeloTAGGG Telomere Length Assay) (Fig. 6D-E; S3 Table). The results from these two assays were in agreement: hESCs in the EcmMech condition showed slightly lower telomerase activity and shortest telomere length compared to the other conditions. We found no significant differences in the percentage of apoptotic cells in the cultures using TUNEL-FACS (Roche) **(** Fig. 6F; S3 Table).

Overall, enzymatic passaging and feeder-free conditions were associated with a statistically significantly higher proliferative rate and a statistically significantly higher percentage of cells in S phase, but compared to the SNP genotyping results, the differences among culture conditions according to these phenotypic assays were relatively minor.

### Assessment of Pluripotency and Differentiation Capacity of hESCs in Different Culture Conditions

The pluripotency of hESCs cultured in the four conditions was assessed by both expression of pluripotency markers and a gene-expression based assay (PluriTest) [21]. Representative images showing the expression of the pluripotency markers OCT4/POU5F1 and SSEA4 by immunocytochemistry are shown in S2A Fig., and indicate that both of these markers were highly expressed in all four conditions after 40 passages. Using PluriTest, we showed that the hESC samples from all four conditions fell well within the pluripotency space and could not be distinguished from each other using this assay (S2B Fig.).

Differentiation capacity was determined using the embryoid body and teratoma assays. Representative images from embryoid body differentiation experiments, demonstrating the ability of cells from each condition to differentiate into every germ layer, are shown in S2C Fig. In the teratoma assays, we quantified the percentage of cells from each germ layer for each tumor (S2D-E Fig.; S4 Table). There were few significant differences in the proportion of derivatives of ectoderm, mesoderm, and endoderm from cells cultured in the different conditions, other than a lower proportion of endodermal derivatives in MefMech compared to EcmMech, and a higher proportion of ectodermal derivatives in EcmEnz compared to MefMech (S2E Fig.; S4 Table).

Staining of teratoma sections with an anti-OCT4 antibody showed that persistence of OCT4 expression varied among the four culture conditions (Fig. 6G-H, S4 Table), with the highest frequency of OCT4-positive foci found in the teratomas from the EcmEnz condition. A low frequency of OCT4-positive foci were detected in teratomas from the MefEnz and EcmMech cultures, and no OCT4-positive foci were found from the MefMech condition. These results mirror the frequency of genetic aberrations found in the different conditions shown in Fig. 2A. We specifically asked whether the number of OCT4-positive foci correlated with deletions of the region of chromosome 17 including the *TP53* gene described above, and found no significant correlation. Persistent OCT4 expression after induction of differentiation is considered to be an undesirable characteristic of in hPSC-derived cultures, as it may indicate a presence of residual pluripotent cells that are resistant to targeted differentiation and have a higher propensity for uncontrolled proliferation. These cells could lead to the generation of malignancies and/or could differentiate into undesired cell types.

### Differences in Genome-wide Gene Expression in hESCs Associated with Time in Culture and Culture Method

We performed genome-wide gene expression profiling of all hESC replicates from the four culture conditions at early (passage 2–6) and late (passage 103–161) passage. We performed multivariate analysis, using time in culture (early vs. late), passage method (enzymatic vs. mechanical), and substrate (MEF vs. ECM). We note that each substrate type was always used with the same medium (MEF with serum replacement medium, and ECM with defined medium).

**Fig 7:**
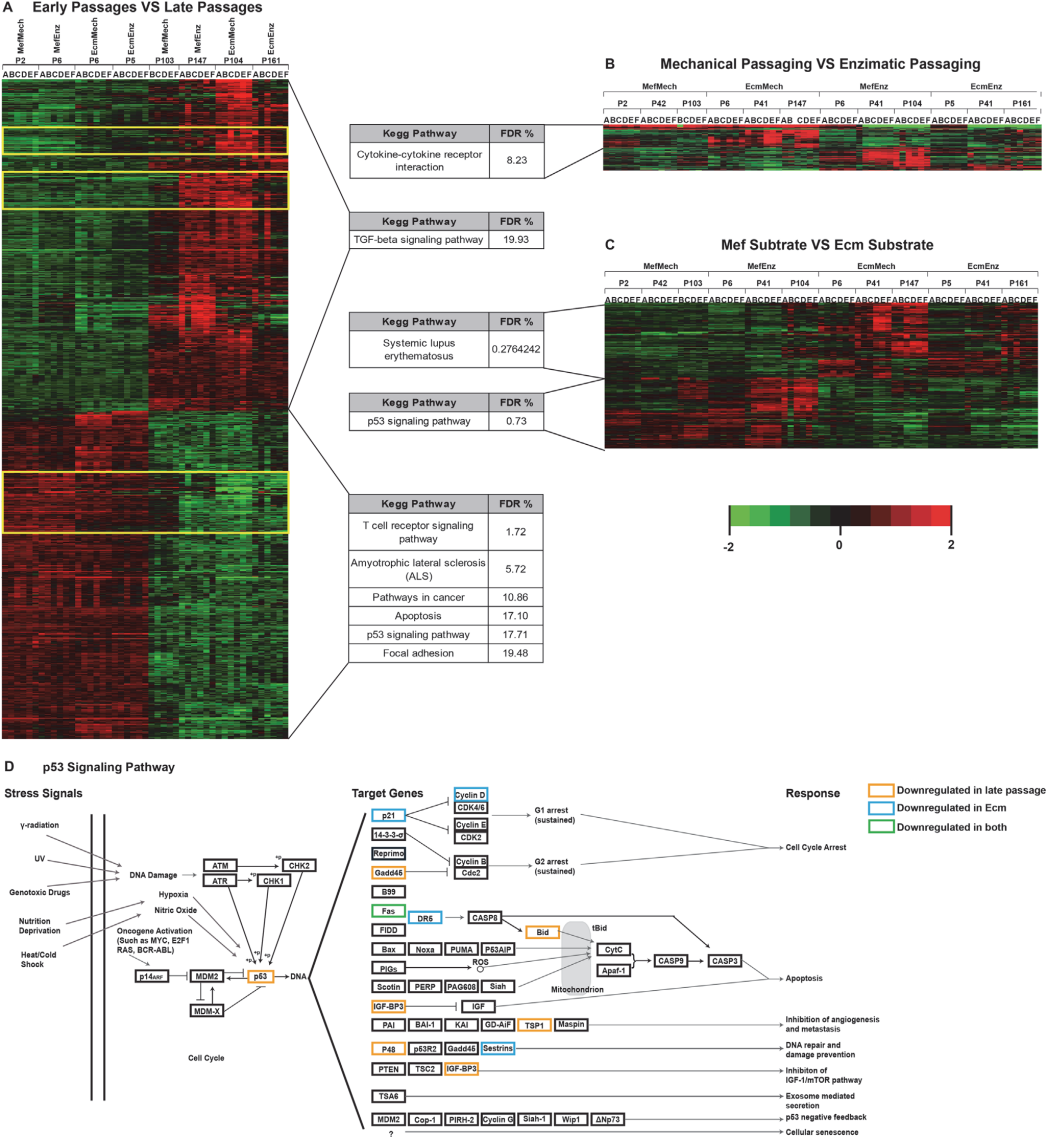
Differences in gene expression with time in culture, passaging method, and media type. Heatmaps showing differentially expressed genes for early vs. late passage **(A)**, mechanical vs. enzymatic passage **(B)**, and Mef vs. Ecm substrate **(C)**. In heatmap **(A)**, the yellow boxes indicate genes for which the expression levels in the late passage MefMech (P103) samples was similar to those in the early passage samples. Probes were selected by multivariate regression. Enrichments in KEGG pathways identified by functional enrichment analysis are shown. Samples were arranged according to passage and culture method, and hierarchical clustering was performed on the genes only. **D**. Diagram of the TP53 signaling pathway, showing genes downregulated in the late vs. early passage samples, and the Ecm vs. Mef substrate comparisons.

We saw significant effects of time in culture on gene expression for all culture conditions (Fig. 7A; S5A Table), although there were three clusters of genes for which the MefMech late passage samples were more similar to the early passage samples (yellow boxes, Fig. 7A). We observed very few effects dependent on the passage method (Fig. 7B; S5C Table) or substrate (Fig. 7C; S5E Table). Pathway analysis, performed on genes differentially expressed over time in culture, using DAVID identified significant changes (FDR <20%) in pathways associated with TP53 signaling, apoptosis, and cancer (Fig. 7A; S5B Table). Mapping the expression levels of the identified genes in the TP53 signaling pathway to a diagrammatic representation of the KEGG pathway, we noted that the expression pattern was consistent with down-regulation in TP53 function over time in culture (Fig. 7D). Inspecting the gene expression levels for these genes at different passages in the four culture conditions, we observed that the differential expression between the early and late passages was most pronounced in conditions carrying a TP53 deletion of in the late passages (MefEnz, EcmMech, and EcmEnz) (S8 Fig.). However, expression changes in the TP53 pathways were also seen in the condition in which deletions in TP53 were not observed (in the MefMech condition), indicating that loss of function of TP53 may occur through epigenetic as well as genetic changes (S3 Fig.).

### Differences in Genome-wide DNA Methylation of hESCs Associated with Time in Culture and Culture Method

In parallel with the gene expression analysis, we performed genome-wide DNA methylation profiling of three hESC replicates from the four culture conditions at early (passages 5–6), mid- (passages 41–42) and late (passages 103–161) passage. We examined X chromosome, imprinted, and non-X/non-imprinted loci separately.

**Fig 8:**
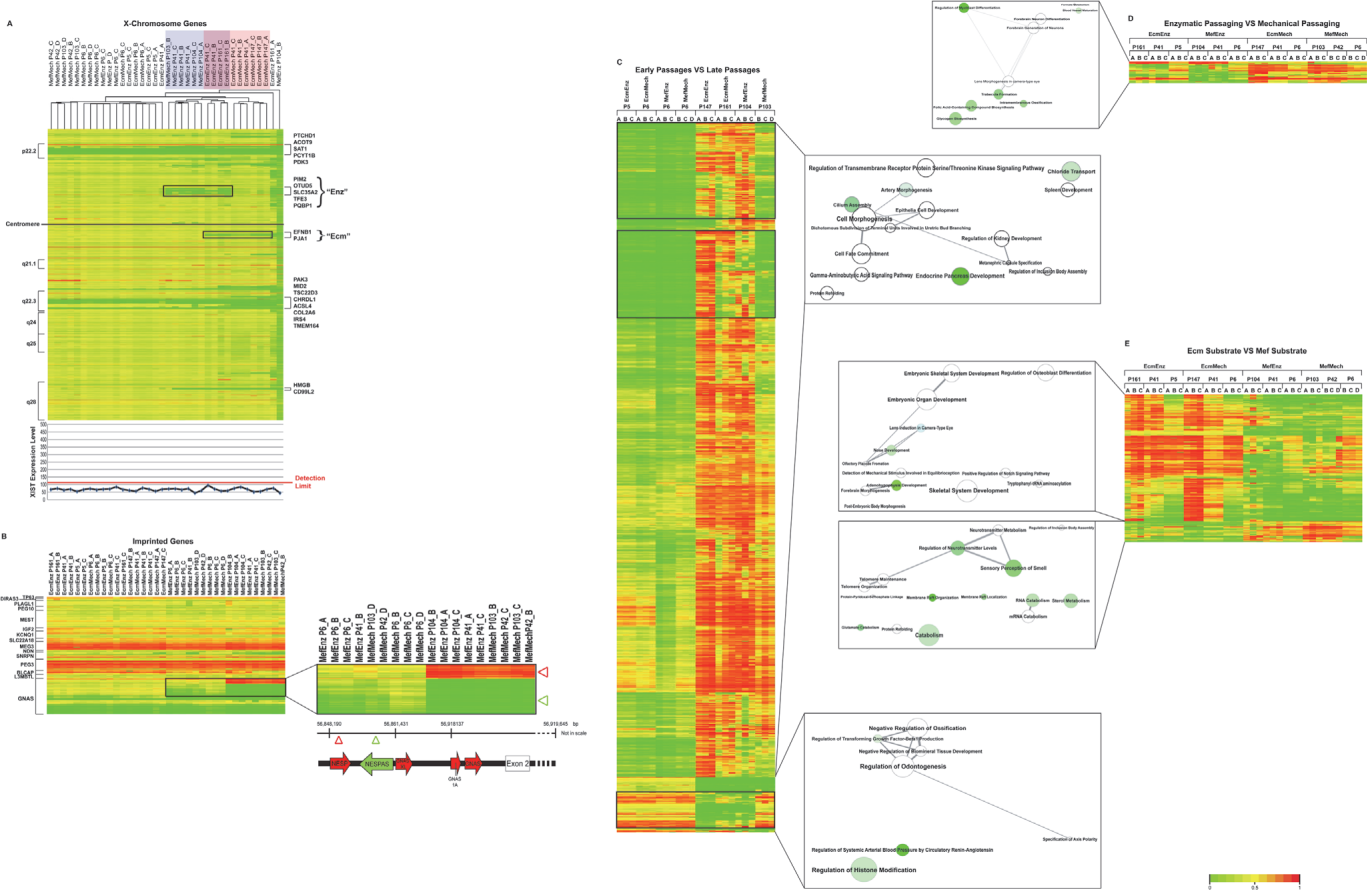
DNA Methylation. **A**. X Chromosome DNA Methylation and XIST Expression. Methylation levels of genes in the X-chromosome (S6A Table) are shown on the heatmap. Hierarchical clustering was performed on the samples, as indicated by the dendrogram. The genes are ordered according to their location (from the beginning to the end of the chromosome). Samples that show loss of DNA methylation for the “Enz” cluster are highlighted in blue, those that show DNA methylation for the “Ecm” cluster are highlighted in pink, and for both clusters in mauve. Genes located in the regions of loss of DNA methylation are listed to the right of the heatmap. XIST expression is shown on the line graph, with the detection limit for the microarray indicated by the red line. **B**. DNA methylation at imprinted loci. Methylation levels for imprinted probes (S6B Table) are shown on the heatmap. Hierarchical clustering was performed on the samples, as indicated by the dendrogram. The genes are ordered according to chromosome location; genes are listed to the left. The inset at the right shows a detail of the NESP/GNAS complex locus, indicating the positions of the CpG sites that were hypermethylated (red triangle) vs. hypomethylated (green triangle) in the late passage samples relative to the NESP/GNAS and NESPAS exons. **C, D, E**. Heatmaps showing differential DNA methylation genes for early vs. late passage **(C)**, mechanical vs. enzymatic passage **(D)**, and Mef vs. Ecm substrate **(E)**. In heatmap **(C)**, the black boxes indicate genes for which the DNA methylation levels in the late passage MefMech (P103) samples was more similar to those in the early passage samples. Probes were selected by multivariate regression. Functional enrichments identified by GREAT analysis are shown to the right of the heatmaps, visualized using REVIGO [13]. Samples were arranged according to passage and culture method, and hierarchical clustering was performed on the genes only. In the functional enrichment results, the size of the node indicated the number of contributing GO terms, and color of the nodes indicates the FDR (darker color for lower FDR), and the edge length indicates the similarity between GO terms (shorter edge for more similar terms).

In earlier studies, we observed that low *XIST* (X (inactive)-specific transcript) expression is associated with variable loss of DNA methylation on the X chromosome, and that approximately half of a large collection of female hPSC lines showed low *XIST* expression, including the WA09 hESC line [22]. In addition, we have observed progressive loss of DNA methylation on the X chromosome with time in culture in hiPSCs that have low *XIST* expression [22]. Consistent with these observations, in the present study there was low expression of *XIST* and focal absence of DNA methylation in several regions of the X chromosome in all conditions at all time-points (Fig. 8A; S6A Table). We also observed expansion of these regions at late passage in some, but not all, conditions. In fact, two regions on the X chromosome showed loss of DNA methylation associated with enzymatic passage (“Enz”) and with extracellular matrix substrate (“Ecm”); we note that the EcmEnz condition samples showed loss of DNA methylation in both of these regions. These results support the notion that the loss of *XIST* expression at early passage is followed by gradual and progressive loss of DNA methylation on the X chromosome, and that this can be influenced by culture conditions.

Genomic imprinting refers to monoallelic expression of specific genomic loci, which is controlled in an epigenetic manner, and we have shown that imprinted gene expression changes with time in culture of hPSCs [22]. Changes in DNA methylation associated with culture condition and time in culture were seen only for the *NESP/GNAS* complex locus, which showed progressive loss of imprinting over time associated with the Mef substrate. Several alternative transcripts arise from the *NESP/GNAS* complex locus due to usage of alternative transcriptional initiation sites and alternative splice sites [23]. The loss of imprinting we observed consisted of hypermethylation in an upstream cluster of CpGs and hypomethylation of a downstream cluster (Fig. 8B; S6B Table).

On a global level, we saw significant effects of time in culture on DNA methylation for all culture conditions (Fig. 8C; S6C Table), although the MefMech late passage samples were similar to the early passage samples. We observed very few effects from passage method (Fig. 8D; S6D Table) or substrate (Fig. 8E; S6E Table). Functional enrichment analysis for sets of DNA methylation probes that showed coherent patterns of methylation was performed using GREAT [17] (Fig. 8C-E, S6F-H Table).

## Discussion

Given the high priority that has been placed on the development of hPSC-based therapies for regenerative medicine, it is critical that we understand and appreciate the impact that the procedures used to manipulate the cells, including derivation, culture, and differentiation, can have on their genetic and epigenetic stability. In this study, we focused on the effects of several common variables during extended culture of hPSCs.

We report the results of a highly replicated study identifying the effects of different substrates and passaging methods over extensive time in culture on genetic and epigenetic stability, and phenotypic characteristics of hPSCs. In both hESCs and hiPSCs, we observed that enzymatic passaging on a feeder-free substrate was associated with an increased accumulation of genetic aberrations compared to mechanical passaging on feeder layers. Long-term culturing of hESCs showed that in the condition associated with the lowest overall rate of genetic aberration (MefMech), the number of aberrations rose significantly only after passage 80 in our study (which corresponded to a total passage number of 80+37 = 117). Detailed experiments in hESCs suggested that the enzymatic passaging had a stronger effect on the accumulation of genetic aberrations compared to the substrate.

We note that these findings may appear to be at odds with a portion of the results from our earlier study, in which we did not observe correlations between passage number or method and the number of aberrations [3]. However, it is important to appreciate the significant differences in the design of the two studies, which are likely to account for the differences in the results. In the earlier study, the hESC analysis was focused on understanding the degree of genetic instability for typical hESC cultures in established stem cell laboratories around the world, and was essentially a cross-sectional survey of hESC cultures from several laboratories, each of which used their own culture and passage methods. Even when there were multiple samples for the same hESC line, they were frequently collected from different labs using different methods at different passage numbers, which may have obscured the effects of the individual variables on genetic stability. In our earlier study, for the hiPSC analysis, the work was largely performed in one laboratory using one set of culture and passage conditions [3]. Although we did not observe a significant difference in the total number of aberrations over time, we did note that deletions tended to occur during reprogramming and early passage, while duplications arose with longer term passage. We saw a similar trend in newly derived hESC lines in a subsequent study performed in collaboration with the laboratory of Dalit Ben-Yosef [24]. In contrast to the earlier study, this is a well-controlled and highly replicated longitudinal study performed in a single lab, in which four hPSC lines were cultured for extended periods of time in four carefully selected combinations of culture and passage conditions. This study design allowed us to observe that the number of CNVs was dependent on passage number, culture condition, and passaging method.

In regard to specific aberrations seen in this study, we observed recurrent duplications of chromosome 12 and chromosome 20q11 in multiple replicates and multiple conditions. Presence of a strong and specific selection pressure for duplications of these regions in hPSCs is supported by several earlier reports showing duplications in the same regions [2–6,10,19]. We saw duplications of chromosome 12 and chromosome 12p alone, but not chromosome 12q alone. In fact, there was one culture in which a duplication of the entire chromosome 12 occurred initially, followed by a loss of the extra copy of 12q. These findings support the conclusion that there was positive selection for a specific sequence on chromosome 12p, and are consistent with our previous results that identified a minimal area of duplication among several different hESC lines that encompassed the expressed *NANOG* pseudogene *NANOGP1* [3]. The identification of the identical relatively small duplication on chromosome 20q11 in multiple replicates of all four culture conditions in this study indicates that this duplication was likely present in a small subset of cells in the original culture, particularly as different duplications encompassing this region have been described in many hPSC lines in prior studies [3,4], and have recently been shown to confer a survival advantage to these cells [7]. Nguyen et al. showed that hESCs with a gain of 20q11.21 had a 2.3-fold increase of Bcl-xL mRNA levels and a 3-fold increase in the protein levels. The mutant hESC cultures underwent 2- to 3-fold less apoptosis upon loss of cell-to-cell contact and were 2-fold more efficient in forming colonies from a single cell when compared with wild type. Transgenic over-expression of BCL2L1 in the wild-type cells led to apoptosis-resistant cells, and BCL2L1-knock-down in the mutant hESC, resulted in a restoration of the wild-type phenotype. This resistance to apoptosis supposes a significant advantage for the mutant cells, explaining the high frequency of gains of 20q11.21 in hPSCs [7].

In terms of cellular phenotype, hESCs cultured in all four conditions were confirmed to be pluripotent by immunocytochemistry for pluripotency markers, the PluriTest assay, *in vitro* differentiation via embryoid body formation, and teratoma formation. Phenotypic assays measuring cell proliferation, telomerase activity and telomere length, and apoptosis showed some statistically significant differences among culture conditions, but these were overall not of practical consequence. We conclude that standard phenotypic assays are not sensitive enough to genetic and epigenetic changes in hESC populations. OCT4 staining of teratoma sections did reveal differences in the frequency of OCT4-positive foci in teratomas generated from cells grown in the different culture conditions, with the highest frequency of OCT4-positive foci from the EcmEnz cultures and no OCT4-positive foci from the MefMech cultures. It is rare for experimental teratomas to retain OCT4-positive undifferentiated cells [25], and it is generally assumed that the retention of undifferentiated cells is an indication of genetic aberrations and that such tumors have a higher potential for metastatic malignancy. Since the EcmEnz condition produced more genetic abnormalities than the other conditions, the presence of undifferentiated cells in the teratomas may be a manifestation of these aberrations.

Multivariate analysis of gene expression and DNA methylation in the hESC cultures showed significant effects from time in culture, with more modest effects attributable to passage method and substrate type. Genes in the *TP53* pathway showed decreased expression with time in culture and in cells cultured on the Ecm substrate, largely reflecting the deletion of the *TP53* gene that occurred at late passage in all conditions except MefMech. The MefMech condition showed the least change in DNA methylation over time in culture, suggesting that this condition may be more favorable for epigenetic as well as genetic stability.

Our results indicate dramatic differences in genetic and epigenetic stability and frequency of persistent foci of OCT4-positive cells after teratoma formation among hPSCs cultured under different conditions. Some, but not all, genetic aberrations we observed included recurrent duplications in genomic regions that we and others have identified as hotspots for duplications in hPSCs in previous studies. Importantly, we observed for the first time recurrent deletion of the genomic region containing the *TP53* gene and consequent loss of expression of the *TP53* transcript in hESCs. *TP53* was first recognized to be a tumor suppressor gene in 1989 [20], and since then, loss of TP53 function has been associated with many cancer types. The high frequency of deletion of genomic regions encompassing the *TP53* gene in hESC cultures suggest that loss of TP53 function gives cells a selective advantage, allowing the abnormal cells to gradually take over the cultures. We note that the deletions observed in our hESCs cultures were single-copy deletions, indicating that even partial loss of TP53 expression may have significant effects. Because loss of *TP53* has been linked to uncontrolled cancerous growth, our observations highlight the need to monitor the genetic stability and expression levels of *TP53* in populations of hPSCs destined for clinical use.

We recognize that use of different enzymatic passaging methods or media formulations may yield different results. Specifically, we performed enzymatic passaging using Accutase, which efficiently dissociates cells into a single-cell suspension, and thus our results may not represent the stability of hPSCs passaged using collagenase or dispase, in which the cells remain in clumps. Moreover, for our feeder-free cultures, we used StemPro media, which may yield different results than the many other media formulations available today. However, our results do conclusively demonstrate that marked differences in genetic stability, and more subtle differences in cellular and molecular phenotype, are seen in hPSC cultures exposed to different passage methods and culture conditions. For hPSC-based cell therapy to be feasible, it will be necessary to produce large quantities of hPSCs, and thus it is notable that we found that two conditions that are commonly used for large-scale expansion of hPSCs, feeder-free culture and enzymatic passaging, were both associated with increased genetic and epigenetic instability. Our study emphasizes the need to meticulously evaluate the effects of new media types, substrates and passaging methods on the stability of hPSC cultures, particularly for cultures intended for clinical use.

## Supporting Information

S1 Fig Gene expression in the chromosome 17 deleted region, as measured by gene expression microarray. Each set of graphs corresponds to one of the genes in the deleted region. Each graph shows the expression level of the respective gene in each condition over time. The red arrows indicate the conditions that carry the deletion. Please note that some probes are located in regions where two or three genes overlap, and that there are two probes each for CD68 and MPDU1.
(TIF)

S2 Fig Analysis of pluripotency and differentiation ability. **A**. Cells cultured in each condition expressed known pluripotency markers OCT4/POU5F1 and SSEA-4 (also stained with DAPI). **B**. The PluriTest algorithm calculates a pluripotency score and a novelty score. Samples for all conditions at early, middle and late passages are indicated by the colored dots. The red cloud indicates the location of the pluripotent cells in the reference data set, and the blue clouds indicate the location of the somatic cells from the reference data set [21]. **C-E**. The differentiation ability of the conditions was shown *in vitro* with embryoid body formation **(C)** and *in vivo* with teratoma formation **(D-E)**. The embryoid bodies were stained with DAPI and the tissue specific markers TUJ1, SOX2, SMA, T-Brachyury, AFP and GATA4. The tissues from the teratomas were identified using hemotoxylin and eosin staining. Percentages of the three germ layers present in the teratomas were assessed visually. Curved lines and stars indicate significant differences in percentages between culture conditions.
(TIF)

S3 Fig Comparing substrates and media via gene expression. Diagram of the TP53 signaling pathway, showing genes that were differentially expressed between the early and late passage time points for each culture method. “Upregulated” indicates genes that were expressed at higher levels at late passage (red), and “downregulated” indicates genes that were expressed at lower levels at late passage (green). **(A)** MefMech, **(B)** MefEnz, **(C)** EcmMech, **(D)** EcmEnz.
(TIF)

S1 Table Tables indicating the samples for the WA09 hESC line (culture condition and passage number) on which each assay type was performed. (XLSX)

S2 Table Lists of all regions of duplication, deletion, LOH and complex CNV found in the samples. **A**. Duplications and complex CNV for the WA09 hESC line. **B**. Deletions and regions of LOH for the WA09 hESC line. **C**. Duplications for the hiPSC lines. **D**. Deletions for the hiPSC lines. **E**. Regions of LOH for the hiPSC lines. The aberrations already present in the control culture (Original_MefMech) are reported separately and were excluded from the lists for each condition. The first column corresponds to the cell line name. Column 2 includes condition name, passage number and replicate. The third column identifies the chromosome in which the aberration occurred. The fourth and fifth columns indicate the start and the end positions of the aberration, respectively. The sixth column represents the copy number for each aberration. The seventh column gives the confidence value of each call. If NA is indicated, it means that the call was manually identified. The eighth column shows the length of the CNV. Comments are written in the ninth column.
(XLSX)

S3 Table Tables indicate the fold change and ANOVA p-values for each of the quantitative phenotypic and differentiation assays. Significant p-values, (less than 0.05), are highlighted in pink.
(XLSX)

S4 Table Histopathologic and immunohistochemistry results from teratoma analysis. For each culture condition, results are listed for all teratomas generated (some injections did not yield teratomas). The cell culture replicate and animal numbers are indicated, as well as the TP53 CNV status (deleted or not deleted). The weight of each teratoma is listed, as well as the % of each section attributable to each germ layer by morphology, and the number of OCT4+ foci observed.
(XLSX)

S5 Table Pathways analysis. Tables **(A, C, E)** show the lists of the gene expression probes that were used for pathways analysis. Lists were derived from multivariate analysis according to the variables time in culture **(A)**, passage method **(C)**, and substrate **(E)**. Tables **(B, D, F)** show the results obtained from the pathways analysis (filtered for FDR>20%) for time in culture **(B)**, passage method **(D)**, and substrate **(F)**.
(XLSX)

S6 Table DNA methylation tables. **A**. List of probes on the X chromosome that were partially methylated in tissue samples and used for Fig. 8A. **B**. List of probes for imprinted genes that were partially methylated in tissue samples and used for Fig. 8B. **Tables (C, D, E)** list probes that have differential DNA methylation according to the variables time in culture **(C)**, passage method **(D)**, and substrate **(E)**. **Tables (F, G, H)** list results from GREAT and REVIGO analyses for time in culture **(F)**, passage method **(G)**, and substrate **(H)**.
(XLSX)
