# Supplementary figures and images for: Increased Risk of Genetic and Epigenetic Instability in Human Embryonic Stem Cells Associated with Specific Culture Conditions
Source: PLoS One. 2015 Feb 25;10(2):e0118307. doi: 10.1371/journal.pone.0118307 (PMC4340884; doi:10.1371/journal.pone.0118307)

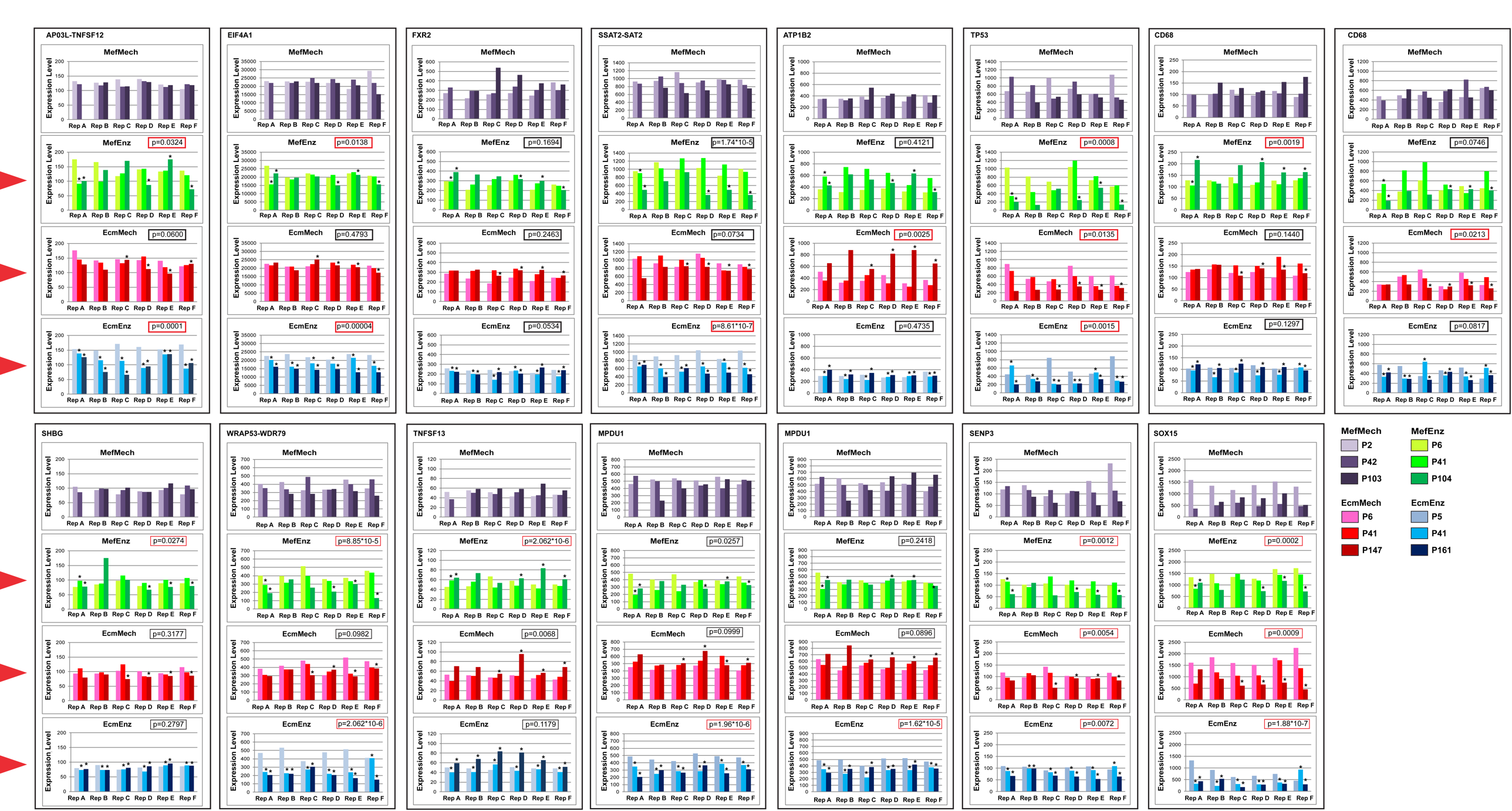

Supplement: S1 Fig — Each set of graphs corresponds to one of the genes in the deleted region. Each graph shows the expression level of the respective gene in each condition over time. The red arrows indicate the conditions that carry the deletion. Please note that some probes are located in regions where two or three genes overlap, and that there are two probes each for CD68 and MPDU1. (TIF) [file pone.0118307.s001.tif]

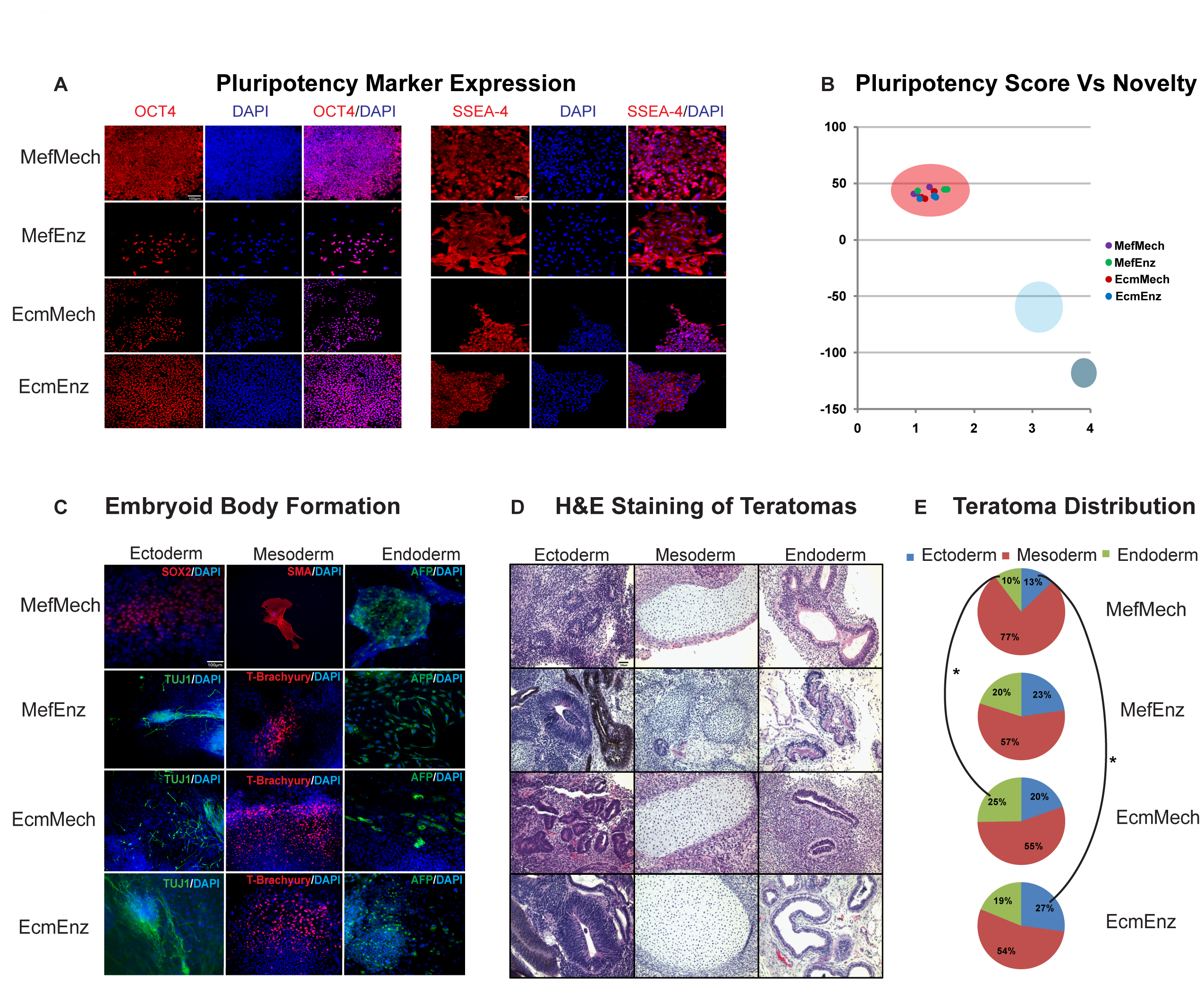

Supplement: S2 Fig — A. Cells cultured in each condition expressed known pluripotency markers OCT4/POU5F1 and SSEA-4 (also stained with DAPI). B. The PluriTest algorithm calculates a pluripotency score and a novelty score. Samples for all conditions at early, middle and late passages are indicated by the colored dots. The red cloud indicates the location of the pluripotent cells in the reference data set, and the blue clouds indicate the location of the somatic cells from the reference data set [21]. C-E. The differentiation ability of the conditions was shown in vitro with embryoid body formation (C) and in vivo with teratoma formation (D-E). The embryoid bodies were stained with DAPI and the tissue specific markers TUJ1, SOX2, SMA, T-Brachyury, AFP and GATA4. The tissues from the teratomas were identified using hemotoxylin and eosin staining. Percentages of the three germ layers present in the teratomas were assessed visually. Curved lines and stars indicate significant differences in percentages between culture conditions. (TIF) [file pone.0118307.s002.tif]

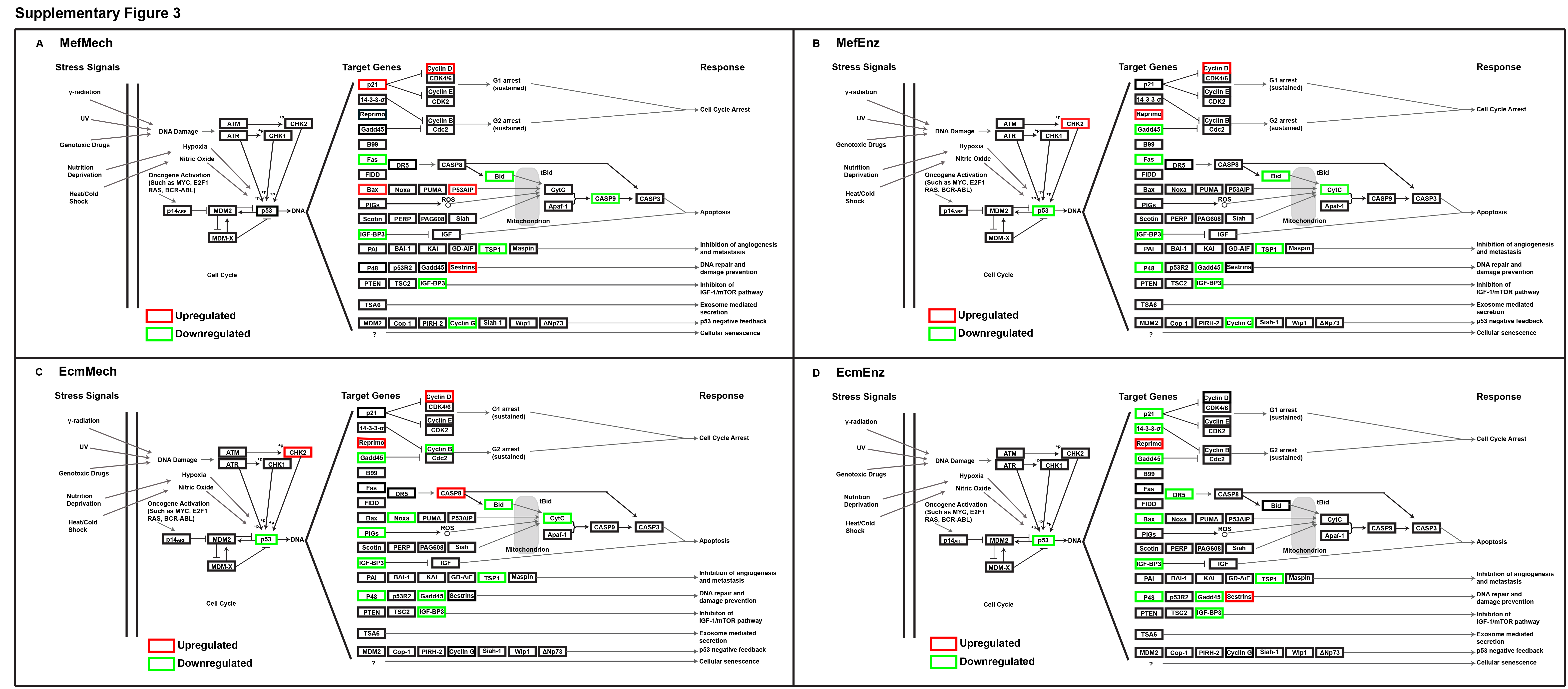

Supplement: S3 Fig — Diagram of the TP53 signaling pathway, showing genes that were differentially expressed between the early and late passage time points for each culture method. “Upregulated” indicates genes that were expressed at higher levels at late passage (red), and “downregulated” indicates genes that were expressed at lower levels at late passage (green). (A) MefMech, (B) MefEnz, (C) EcmMech, (D) EcmEnz. (TIF) [file pone.0118307.s003.tif]
